# The effects of public health policies on population health and health inequalities in European welfare states: protocol for an umbrella review

**DOI:** 10.1186/s13643-016-0235-3

**Published:** 2016-04-08

**Authors:** Katie Thomson, Clare Bambra, Courtney McNamara, Tim Huijts, Adam Todd

**Affiliations:** Centre for Health and Inequalities Research, Department of Geography, Durham University, Lower Mountjoy, South Road, Durham, DH1 3LE UK; Department of Sociology and Political Science, Norwegian University of Science and Technology, Dragvoll, Building 9, Level 5, 7491 Trondheim, Norway; Centre for Primary Care and Public Health, Barts and The London School of Medicine and Dentistry, Yvonne Carter Building, 58 Turner Street, Whitechapel, London, E1 2AB UK; Division of Pharmacy, School of Medicine, Pharmacy and Health, Durham University, Stockton-on-Tees, TS17 6BH UK

**Keywords:** Public health, Health and health inequalities, Europe, Welfare states, Umbrella systematic review

## Abstract

**Background:**

The welfare state is potentially an important macro-level determinant of health that also moderates the extent, and impact, of socio-economic inequalities in exposure to the social determinants of health. The welfare state has three main policy domains: health care, social policy (e.g. social transfers and education) and public health policy. This is the protocol for an umbrella review to examine the latter; its aim is to assess how European welfare states influence the social determinants of health inequalities institutionally through public health policies.

**Methods/design:**

A systematic review methodology will be used to identify systematic reviews from high-income countries (including additional EU-28 members) that describe the health and health equity effects of upstream public health interventions. Interventions will focus on primary and secondary prevention policies including fiscal measures, regulation, education, preventative treatment and screening across ten public health domains (tobacco; alcohol; food and nutrition; reproductive health services; the control of infectious diseases; screening; mental health; road traffic injuries; air, land and water pollution; and workplace regulations). Twenty databases will be searched using a pre-determined search strategy to evaluate population-level public health interventions.

**Discussion:**

Understanding the impact of specific public health policy interventions will help to establish causality in terms of the effects of welfare states on population health and health inequalities. The review will document contextual information on how population-level public health interventions are organised, implemented and delivered. This information can be used to identify effective interventions that could be implemented to reduce health inequalities between and within European countries.

**Systematic review registration:**

PROSPERO CRD42016025283

**Electronic supplementary material:**

The online version of this article (doi:10.1186/s13643-016-0235-3) contains supplementary material, which is available to authorized users.

## Background

Socio-economic inequalities are associated with unequal exposure to social, economic and environmental risk factors, which in turn contribute to health inequalities. People with higher income, employment and educational opportunities have lower mortality and morbidity [1]. Social inequalities in health are widespread, for example in Europe where an estimated 80 million people are living in relative poverty [2]. Important European differences in health outcomes have been attributed to variations in how the welfare state is administered [3, 4]. The welfare state is therefore potentially an important macro-level determinant of health which also moderates the extent, and impact, of socio-economic inequalities in exposure to the social determinants of health. The welfare state has three main policy domains: health care, social policy (e.g. social transfers and education) and public health policy. This planned umbrella review examines the latter; its aim is to assess how European welfare states influence the social determinants of health inequalities institutionally through public health policies. Understanding the impact of specific public health policy interventions will help to establish causality in terms of the effects of welfare states on population health. This review will therefore help identify effective interventions that could be implemented to reduce health inequalities between and within European countries.

Many commentators have sought to define what is meant by public health. The World Health Organization [5] emphasises how public health refers to ‘all organized measures (whether public or private) to prevent disease, promote health, and prolong life among the population as a whole’. The system of administering public health to populations could be the private or voluntary sector, but in European welfare states, it is most usually instigated by governments—centrally, regionally or locally. Welfare states may impact the health of citizens either indirectly through influencing the social determinants of health (e.g. through changes to social policy such as education, social security and housing) or directly through health care systems or policies aimed at promoting public health specifically [6, 7]. The proposed umbrella review will examine the latter aspect of European welfare states.

Public health policies can operate on a number of different levels, which affects population health and health inequalities. Following Mackenbach and McKee [8], public health policies may influence primary prevention (which aims to avoid the occurrence of a disease by reducing exposure to health risks) or secondary prevention (which aims to avoid the development of a disease to a symptomatic stage by diagnosing and treating the disease before it causes significant morbidity of the disease) (p. 195). Public health interventions may occur at multiple levels. Downstream interventions involve individual-level behavioural approaches for prevention or disease management, and their success depends on whether some sections of the population are more likely to take up or successfully engage with certain initiatives compared to others [9]. Upstream interventions involve state or institutional control, regulating the supply of a particular substance or activity, promoting a method of preventative health behaviour or improving the wider environment. These population-level interventions will be the focus for the proposed umbrella review, as they are likely to reduce socio-economic inequalities in health and have the greatest influence on overall population health within a territory [10–13].

The nature of public health interventions means their influence percolates into many aspects of how we behave, live and work. For the purposes of this review, we categorise these interventions into fiscal policy, regulation, education, preventative treatment and screening. It is also helpful to consider the broad areas by which local and national governments may intervene and regulate. An example of the public health domain groupings (and intervention types) we propose can be found in Table 1. These groups are based on the ten areas of public health policy that Mackenbach and McKee [7, 14] identify as contributing to major population health gains: tobacco; alcohol; food and nutrition; reproductive health services; the control of infectious diseases; screening; mental health; road traffic injuries; air, land and water pollution; and workplace regulations. Whilst acknowledging that this list may not be exhaustive, its inclusion highlights the broad areas that our final report will focus on. Furthermore, distinguishing public health policies from other welfare state policy domains such as social policy may not be clear-cut (the division is based on practicality as a parallel review on social and health care policy is also underway). Public health policies influence almost all aspects of society, but the focus here centres on policies directly influencing health (e.g. the control of infectious diseases), or those indirectly regulating other areas of government regulation policy which have clear and direct pathways to (poor) health (e.g. workplace regulations).

**Table 1:** Matrix of population-level preventative public health interventions

Prevention type		Primary prevention			Secondary prevention	
Type of intervention		Fiscal measures	Regulation	Education, communication and information	Preventative treatment	Screening
Description		Using market forces to change demand for products deemed healthy/unhealthy	Making and enforcing regulation to encourage/discourage products and services deemed healthy/unhealthy	Using mass media campaigns to encourage/discourage products and services deemed healthy/unhealthy	Offering population-wide measures to eradicate infectious diseases	Offering age-appropriate population-level screening for certain diseases
Domains	Scope of domains					
Tobacco	Protecting people from second-hand smoke and raising tobacco prices through taxation	✓	✓	✓		
Alcohol	Increasing the price limits of alcohol and availability and bans on advertising	✓	✓	✓		
Food and nutrition	Regulating supplements of trace minerals (e.g. iodine and fluoride) and tackling nutrition-related risk factors of cardiovascular diseases	✓	✓	✓		
Reproductive health services	Fertility (access to contraception and safe abortion, prevention of multiple births in assisted reproduction), pregnancy (protection of pregnant women and children, preventive care in the prenatal period, screening for congenital anomalies), delivery and postpartum care (access to safe delivery care, promotion of breastfeeding)	✓	✓	✓		✓
The control of infectious diseases	Protecting the health of the public from new or persisting threats, securing what has been achieved (e.g. system breakdown during economic crises or methicillin-resistant *Staphylococcus aureus*).		✓	✓	✓	
Screening	Cancer screening (cervical, breast, colorectal and prostate screening, etc.) and screening for CVD risk factors (e.g. hypertension prevention and control)			✓		✓
Mental health	The human rights perspective, scope of mental health policy, intervening with those at risk, intervening with the process of suicide		✓	✓		
Road traffic injuries	Controlling speed, stopping driving when under the influence of alcohol, enforcing use of safety equipment, increasing conspicuousness, improving vehicle crash protection, making infrastructural changes to road design		✓	✓		
Air, land and water pollution	Effectiveness of air pollution control policies (sulphur dioxide, particulate matter, nitrogen oxides, ozone). Land and water pollution control policies such as land decontamination		✓	✓		
Workplace regulations	Working week regulations, workplace health and safety legislation (e.g. around exposures to noise and vibrations)		✓	✓		

Whilst there are many excellent reviews which focus on specific public health areas (e.g. [15, 16]), to our knowledge, there is no truly comprehensive umbrella systematic review which has sought to evaluate the full suite of population-level public health policies available to governments. Lorenc et al. [10] undertook a rapid review searching only one database (Medline) and identified 12 reviews meeting their inclusion criteria. Bambra et al. [17] conducted a much more complete review which focused on both social and public health policies. However, their searching only spanned the period 2000–2007, and at that time, the authors concluded that the systematic review evidence base was unclear to determine the effects of interventions on health inequalities. Nor did these previous reviews focus on the potential importance of different welfare state context. In recent years, there has been an effort to promote health equity by encouraging authors of systematic reviews to document health inequalities amongst disadvantaged groups through reporting guidelines such as ‘PRISMA-E 2012’ and ‘PROGRESS-PLUS’ [18–20]. It is therefore timely to update these umbrella reviews and comprehensively document population-level public health interventions designed to improve health and reduce health inequalities.

## Methods

Our systematic review was designed using the Preferred Reporting Items for Systematic review and Meta-Analyses (PRISMA) guidelines [21]. A PRISMA-P checklist is available as an Additional file 1 to this protocol. This protocol is registered with PROSPERO (CRD42016025283).

### Research question

What are the effects of population-level public health policies on health and health inequalities in European welfare states?

### Study design

A systematic review methodology will be used to locate and evaluate published systematic review-level evidence on the effects of public health policy regulation on health and inequalities in health (‘umbrella review’) [17, 22, 23]. Umbrella reviews are an established method of locating, appraising and synthesising systematic reviews [24]. Umbrella reviews are therefore able to present the overarching findings of such systematic reviews (usually considered to be the highest level of evidence) and can also extract data from the best quality studies within them [17]. In this way, they represent an effective way of rapidly reviewing a broad evidence base. An umbrella review methodology is an increasingly used technique in public health and medical research but is seldom used in the evaluation of institutional policies or the social determinants of health [24, 25]. Although umbrella reviews have been published on particular aspects of public health interventions (e.g. [13, 15, 17, 22]), no comprehensive umbrella review has been reported detailing the full suite of public health policies which governments may use to influence public health and reduce health inequalities.

### Inclusion criteria

Following standard evidence synthesis approaches [18], the inclusion criteria for the review are determined a priori in terms of PICOS (Population, Intervention, Comparison, Outcome and Setting; [26]).

- *Population*: Children and adults (all ages) in any high-income country (defined as Organisation for Economic Co-operation Development (OECD) members) and additional EU-28 members not OECD members.^1^ The population is kept purposively broad to allow the widest range of literature to be identified.
- *Intervention*: Upstream, population-level and public health policies defined as primary and secondary interventions. The inclusion criteria are purposely broad to allow for a range of different public health interventions to be located. Table 1 gives an indication of the type of interventions which this review may highlight. The domains listed and the specific intervention types are however illustrative of the variety of policy areas and interventions which public health spans and should not be considered exhaustive.
- *Comparison*: We will include systematic reviews that include studies with and without controls. Acceptable controls include randomised or matched designs.
- *Outcomes*: Health and health inequality outcomes. Primary outcome measures include (but are not limited to) morbidity, health behaviours, mortality, accidents and injuries. Secondary outcomes relate to health inequalities in terms of gender, ethnicity and socio-economic status (defined as individual income, wealth, education, employment or occupational status, benefit receipt; as well as area-level economic indicators). When available, cost-effectiveness data will also be collected.
- *Setting*: Only systematic reviews will be included in the analysis.

Following the methods of previous umbrella reviews [17, 22], publications will need to meet the two mandatory criteria of Database of Abstracts of Reviews of Effects (DARE): (i) that there is a defined review question (with definition of at least two of the participants, interventions, outcomes or study designs) and (ii) that the search strategy included at least one named database, in conjunction with either reference checking, hand searching, citation searching or contact with authors in the field. When two reviews are identified with the same research question, only the most recent umbrella review will be synthesised as part of this study. A rigorous and inclusive literature search for existing systematic reviews will be conducted, incorporating a range of study designs (following [27]), including randomised and nonrandomised controlled trials, randomised and nonrandomised cluster trials, prospective and retrospective cohort studies (with and/or without control groups), prospective repeat cross-sectional studies (with and/or without control groups) and interrupted time series (with and/or without control groups).

### Search strategy

Twenty databases will be searched from their start until March 2016 (host sites given in parentheses): Medline (Ovid), Embase (Ovid), Cumulative Index to Nursing and Allied Health Literature (CINAHL; EBSCOhost), PsycINFO (EBSCOhost), Social Science Citation Index (Web of Science), Applied Social Sciences Index and Abstracts (ASSIA; ProQuest), International Bibliography of the Social Sciences (IBSS; ProQuest), Sociological Abstracts (ProQuest), Social Services Abstracts (ProQuest), PROSPERO (Centre for Reviews and Dissemination, University of York), Campbell Collaboration Library of Systematic Reviews (The Campbell Library), Cochrane Library (includes Cochrane Database of Systematic Reviews, Cochrane Central Register of Controlled Trials, Cochrane Methodology Register, DARE, Health Technology Assessment Database, NHS Economic Evaluation Database; Wiley), Database of Promoting Health Effectiveness Reviews (DoPHER; EPPI-Centre), Social Care Online (SCIE) and Health Systems Evidence. All searches will be tailored to the specific host site; an example search strategy is shown for Medline in Additional file 2. To complement these searches, citation follow-up from the bibliographies and reference lists of all included articles will be conducted. No language or publication date restrictions will be included. Searches will be limited to peer-reviewed publications only. Authors will be contacted to obtain any relevant information that is missing. If reviews do not have sufficient data, they will be excluded from further analysis.

The proposed search terms used in the search strategy are shown in Additional file 2. After careful consideration, and some initial searches, inequality terms were not included in the final search strategy. It was decided to screen the articles after the initial search to maximise ‘hits’ using the PROGRESS-Plus acronym recommended by the Cochrane/Campbell Health Equity Group [18, 19]. The framework includes socio-economic factors that may impact health equity including Place of residence, Race/ethnicity/culture/language, Occupation, Gender/sex, Religion, Education, Socio-economic status and Social capital [28]. The additional ‘Plus’ captures further variables of age, disability and sexual orientation that may indicate a disadvantage [18]. Due to the diverse nature of interventions this review will synthesise, a discrete list of health outcomes has not been generated either, but will be reviewed post screening.

### Screening, data extraction and quality appraisal

The initial screening of titles and abstracts using EndNote will be conducted by one reviewer (KT), with a random sample of at least 10 % (in keeping with previous successful reviews, e.g. [27]) checked by a second reviewer (AT or CM). Full-text copies of potentially relevant articles will then be examined for inclusion by two reviewers independently (KT and CM or AT). Any discrepancies will be resolved through discussion between the two reviewers, and, if consensus is not reached, with the project lead (CB). Furthermore, inter-rater reliability will be assessed using the kappa statistic.

Data will be extracted using standard data extraction forms based on previous reviews [17]. The following data will be extracted: the intervention type reviewed; the study population in the review (and in the included studies); any age/gender/location, etc. restrictions in the review; the number of relevant studies in the review (total); number of databases searched (total); whether grey literature was searched or citation follow-up conducted; any time/language/country restrictions in the review; study design of studies included in the review (e.g. randomised controlled trials (RCTs), controlled prospective cohort, repeat cross sections); the method of synthesis (meta-analysis or narrative); any details on implementation of interventions contained within the review; and the main findings both at a population level and in terms of socio-economic inequalities in health.

Quality will be assessed using a checklist adapted from DARE, which has been used successfully in previous umbrella reviews [22]. Articles will be categorised as low (met 0–3 criteria), medium (4–5) or high (6–7) quality, with one point attributed for each of the questions answered ‘yes’ on the methodological checklist in Table 2.

**Table 2:** Methodological quality checklist

1. Is there a well-defined question?
*The question should define at least the participants, the intervention, outcomes and the study designs.*
2. Is there a defined search strategy?
*The search strategy should include at least one named database combined with reference checking, hand searching, citation follow-up or expert contact.*
3. Are inclusion/exclusion criteria stated?
*The review should make the grounds for study inclusion and exclusion transparent in terms of participants, interventions, outcomes and study designs.*
4. Are study designs and number of studies clearly stated?
*The review should outline the designs of included studies and make it clear which and how many studies are in the final synthesis.*
5. Have the primary studies been quality assessed?
*The quality assessment process should be transparent in the review and should clearly describe which quality appraisal tool is used, and the relative quality of the included study.*
6. Have the studies been appropriately synthesised?
*The review should use either meta-analysis or narrative synthesis depending on the heterogeneity and methodological quality.*
7. Has more than one author been involved in each stage of the review process?
*To minimise bias, at least two reviewers should be involved in each stage of the review process (study selection, data extraction, quality appraisal, synthesis).*

Source: Adapted from [13, 17, 22, 36, 37]

### Synthesis

If meta-analysis has been undertaken, the effect size will be used. In cases of narrative summaries where no summary effect sizes are provided, an exploration of patterns in the data will be accompanied by a discussion of similarities and differences between the findings of different studies. A detailed commentary on the major methodological problems or biases in the review will also be included, alongside an assessment of completeness and applicability [29]. We will also incorporate an assessment of the quality of included systematic reviews in our interpretation of findings—something which has been lacking in previous umbrella reviews [25]. We will synthesise the health effects at a population level and also at subgroup level with regard to health inequalities (e.g. gender, ethnicity and socio-economic status). An assessment of the strength of evidence will be made using GRADE [30].

## Pilot search strategy

A pilot search strategy has been conducted in Medline (via Ovid) and is shown in Table 3. At each stage, the type of study (pilot 1), intervention (pilot 2) and outcomes (health, pilot 3 and SES, pilot 4) are added. Three key papers were used as examples to see if the different searches located them. Pilot search 1 used a search strategy based primarily on the Health Information Research Unit of McMaster University [31] and also the Scottish Intercollegiate Guidelines Network (SIGN) filter for systematic reviews [32]. Additionally, specific reference to umbrella reviews was included to ensure existing umbrella systematic reviews were highlighted and their bibliographic literature added where necessary. This identified over 355,412 records. Next, population-level intervention terms were added (pilot 2). When combined with the systematic review terminology previously searched, the number of hits dropped dramatically to 8,821. Pilot 3 includes examples of outcomes and reduces the number of hits only slightly to 8,550. Adding inequality terminology reduced the number of hits further to ca. 1,700 (pilot 4). Although adding outcome terms (pilots 3 and 4) decreased the number of hits to one fifth compared to just using the type of study and population-level terms (pilot 2), it was felt that these outcome terms should not be included in the final search strategy. Instead, the search strategy advocated in pilot 2 would be used and screening for outcome terms would occur after the initial searches have been conducted. This was in part due to the variety of interventions (and therefore outcomes) which this public health review might highlight. The search strategy will be adapted for each of the specific databases; an example for Medline (Ovid) is shown in Additional file 2.

**Table 3:** Pilot search strategy using Medline (via Ovid), run from start date to present (11/03/2016)

	Study design	Intervention: population level	Outcomes: health related	Outcomes: inequalities
Search number	1	2	3	4
Search strategy reference (including deviations)	Terms from McMaster University [31] and SIGN [32] (plus specific umbrella review terminology)	Terms from Bambra et al. [27] (changed positional operator (adj) from 3 to 8 and included additional search term using health adj8 to intervention terminology)	Terms from Cairns et al. [22]	Terms from Bambra et al. [17] (excluding fluoridation and water supply, access to health care, public transport and neighbourhood crime terminology)
Search strategy details in Additional file 2 including deviations from	Lines 1–6 (excluding animal studies—lines 11–13)	Lines 7 to 10	Lines 15 and 16	Lines 17 and 18
Number of hits	355,412	8,821	8,550	1,724
Target papers				
Bambra et al. [17]	✓	✓	✓	✓
Main et al. [13]	✓	✓	✓	✓
Oldroyd et al. [35]	✓	✓	✓	✓

The complete search strategy is detailed in Additional file 2 (online)

## Discussion

This umbrella review will provide evidence of macro, population-level public health interventions which affect health and reduce health inequalities amongst European welfare states. Understanding the impact of specific public health policy interventions will help to establish causality in terms of the effects of welfare states on population health and health inequalities and, most importantly, identify effective interventions that could be implemented to reduce health inequalities across European countries. The umbrella review will consider public health strategies across ten different domains of public health, and, as such, it will also serve as a mapping exercise of the types of interventions that have been systematically reviewed, thereby highlighting any gaps in the systematic review evidence base. The review will also seek to establish (where reported) how such public health interventions are organised, implemented and delivered. Context is increasingly recognised as an important factor in the success of public health interventions [33] and has begun to be taken into account in systematic reviews. However, the assessment of implementation has not featured strongly in previous umbrella reviews. We will therefore develop and refine existing methodological tools and apply them to umbrella reviews [33, 34]. The review also adds to the literature that conceptualises public health regulation as one of the three tiers of the welfare state—alongside health care access/provision and social policy [14].

## Additional files

Additional file 1: PRISMA-P (Preferred Reporting Items for Systematic review and Meta-Analyses Protocols) 2015 checklist: recommended items to address in a systematic review protocol. (DOC 83 kb) Additional file 2: Search Strategy—Medline (Ovid). (DOC 39 kb)

## Abbreviations

DARE: Database of Abstracts of Reviews of Effects
EU-28: European Union member countries
PICOS: Population, Intervention, Comparison, Outcome and Setting
PRISMA: Preferred Reporting Items for Systematic Reviews and Meta-Analyses
PROGRESS-Plus: Place of residence, Race/ethnicity/culture/language, Occupation, Gender/sex, Religion, Education, Socio-economic status and Social capital-Plus
PROSPERO: international prospective register of systematic reviews
RCT: randomised controlled trial

## Footnotes

1 The World Bank classifies as high-income countries those countries with GNI per capita income of $12,736 or more for the current 2016 fiscal year. Further details can be found at http://data.worldbank.org/income-level/OEC. The list of OECD countries includes Australia, Austria, Belgium, Canada, Chile, Czech Republic, Denmark, Estonia, Finland, France, Germany, Greece, Hungary, Iceland, Ireland, Italy, Israel, Japan, Korea Republic, Luxembourg, Netherlands, New Zealand, Norway, Poland, Portugal, Slovak Republic, Slovenia, Spain, Sweden, Switzerland, UK and the USA. Additional EU-28 countries not included in the previous list were also added (including Bulgaria, Croatia, Cyprus, Latvia, Lithuania, Malta and Romania).
