# Supplementary material for: The effects of public health policies on population health and health inequalities in European welfare states: protocol for an umbrella review
Source: Syst Rev. 2016 Apr 8;5:57. doi: 10.1186/s13643-016-0235-3 (PMC4826536; doi:10.1186/s13643-016-0235-3)
Supplement: Additional file 2: — Search Strategy—Medline (Ovid). (DOC 39 kb) [file 13643_2016_235_MOESM2_ESM.doc]

**Search Strategy – MEDLINE (Ovid)**

Lines 1 to 14 (in black) detail the final search strategy which will be used for the review. The remaining searches in **blue**, detail the full pilot search strategy where:

Lines 15 and 16 detail the health-related outcomes.

Lines 17 and 18 detail the inequality outcomes.

The pilot search strategy was run on the 10^th^ March 2016 using Ovid Medline (in-process and other non-indexed citations and Ovid Medline 1946 to present).

| 1 | search*.tw. |
| --- | --- |
| 2 | (systematic adj2 (review* or overview*)).mp. or Systematic Review/ |
| 3 | (meta analy* or metaanaly*).mp. or Meta Analysis/ |
| 4 | (umbrella adj2 review).tw. |
| 5 | "review of reviews".tw. |
| 6 | 1 or 2 or 3 or 4 or 5 |
| 7 | (("population level" or "population based" or "population orientated" or "population oriented" or "community level" or "community based" or "community orientated" or "community oriented") adj8 (intervention$ or prevention or policy or policies or program$ or project$)).tw. |
| 8 | (health adj8 (intervention$ or prevention or policy or policies or program$ or project$)).tw. |
| 9 | 7 or 8 |
| 10 | 6 and 9 |
| 11 | animals/ |
| 12 | humans/ |
| 13 | 11 not (11 and 12) |
| 14 | 10 not 13 |
| 15 | (health or death or mortality or disease or ill$ or morbidity or injur$ or accident$ or casualt$).tw. |
| 16 | 14 and 15 |
| 17 | (inequality or inequalities or equality or inequity or inequities or equity or disparity or disparities or gap or gaps or gradient or gradients or unequal or disadvantage$ or variation$ or socioeconomic or adult educat$ or lifelong learn$ or life long learn$ or adult literacy or university educat$ or educational level$ or educational attain$ or education$ empower$ or social mobility or widen$ participat$ or unemployment or workless$ or jobless$ or income support or jobseekers allowance or employment status or full employment or labour market polic$ or labour market polic$ or vocational train$ or vocational education or vocational rehabilitation or economic activity or welfare to work or new deal or poverty or low income or social security benefit$ or standard of living or minimum wage$ or tax credit$ or minimum salar$ or welfare benefit$ or poverty trap or debt trap or tax rebate$ or income support or deprivation or social exclusion or social inclusion).tw. |
| 18 | 16 and 17 |
